# Ready, set, go: a cross-sectional survey to understand priorities and preferences for multiple health behaviour change in a highly disadvantaged group

**DOI:** 10.1186/s12913-016-1701-2

**Published:** 2016-09-13

**Authors:** Natasha Noble, Christine Paul, Robert Sanson-Fisher, Heidi Turon, Nicole Turner, Katherine Conigrave

**Affiliations:** 1 Priority Research Centre for Health Behaviour, School of Medicine and Public Health, University of Newcastle, Callaghan, NSW 2308 Australia; 2 School of Medicine and Public Health & Department of Rural Health, University of Newcastle, Callaghan, NSW 2308 Australia; 3 Sydney Medical School, University of Sydney, Sydney, NSW 2000 Australia

**Keywords:** Lifestyle risk factors, Health behaviour change, Readiness to change, Social disadvantage, Aboriginal Australians

## Abstract

**Background:**

Socially disadvantaged groups, such as Aboriginal Australians, tend to have a high prevalence of multiple lifestyle risk factors, increasing the risk of disease and underscoring the need for services to address multiple health behaviours. The aims of this study were to explore, among a socially disadvantaged group of people attending an Aboriginal Community Controlled Health Service (ACCHS): a) readiness to change health behaviours; b) acceptability of addressing multiple risk factors sequentially or simultaneously; and c) preferred types of support services.

**Methods:**

People attending an ACCHS in regional New South Wales (NSW) completed a touchscreen survey while waiting for their appointment. The survey assessed participant health risk status, which health risks they would like to change, whether they preferred multiple health changes to be made together or separately, and the types of support they would use.

**Results:**

Of the 211 participants who completed the survey, 94 % reported multiple (two or more) health risks. There was a high willingness to change, with 69 % of current smokers wanting to cut down or quit, 51 % of overweight or obese participants wanting to lose weight and 44 % of those using drugs in the last 12 months wanting to stop or cut down. Of participants who wanted to make more than one health change, over half would be willing to make simultaneous or over-lapping health changes. The most popular types of support were help from a doctor or Health Worker and seeing a specialist, with less than a quarter of participants preferring telephone or electronic (internet or smart phone) forms of assistance. The importance of involving family members was also identified.

**Conclusions:**

Strategies addressing multiple health behaviour changes are likely to be acceptable for people attending an ACCHS, but may need to allow flexibility in the choice of initial target behaviour, timing of changes, and the format of support provided.

**Electronic supplementary material:**

The online version of this article (doi:10.1186/s12913-016-1701-2) contains supplementary material, which is available to authorized users.

## Background

### Impact of lifestyle risk factors on chronic disease and mortality

Lifestyle risk factors such as smoking, poor nutrition, physical inactivity and excess alcohol are among the leading causes of mortality and disease worldwide [1, 2]. These risk factors tend to be more prevalent among socially disadvantaged and indigenous groups [3–5], for a range of complex cultural and historical reasons [6]. Aboriginal Australians are one example of a socially disadvantaged group for whom key lifestyle changes such as smoking cessation, increased physical activity, reduced alcohol intake and improved nutrition are needed to achieve health equality with the mainstream Australian population [7, 8].

### Clustering of health risk behaviours

Health risk behaviours tend to co-occur or cluster together [9–12] with individuals rarely meeting guidelines across multiple behaviours [13]. Socially and economically disadvantaged populations are likely to exhibit a greater number of lifestyle risk factors [14, 15]. For example, Aboriginal Australians visiting a general practitioner were four times more likely to be overweight, daily smokers *and* to consume alcohol at risky levels than other patients [16]. With the rising burden of chronic and cardiovascular diseases worldwide, there is growing recognition that multiple risk factor intervention should be the cornerstone of primary prevention [14]. The clustering of risk factors among socially disadvantaged groups has particular implications for the workload of primary care teams in deprived areas [14].

### Multiple health behaviour change interventions

There is limited but growing evidence supporting the effectiveness of multiple behaviour change interventions [17]. Intervention studies aimed at both diet and physical activity have shown significant improvements for both behaviours [18], while tailored advice for five behaviours including physical activity, fruit, vegetable and fat intake, and smoking cessation was effective in improving dietary behaviours and physical activity, but not smoking [19]. Women who were successful in increasing exercise levels also showed increased efforts to quit smoking [20].

However, as an emerging area of public health research, much remains unknown about multiple health behaviour change, such as the optimal number of behaviours with which to intervene, whether to intervene simultaneously or sequentially, and how to achieve synergies to improve multiple behaviours [21]. A critical issue for health services is the acceptability and effectiveness of multiple behaviour change approaches to care. The potential for multiple behaviour change interventions to overwhelm or discourage participants [22, 23] may be a particular issue for socially disadvantaged populations generally, and for Aboriginal Australians in particular, given that the latter experience higher levels of stressful life events and general psychological distress compared to other Australians [24, 25].

### Need for culturally targeted approaches to improving health for disadvantaged and indigenous groups

Understanding consumer perspectives is critical to the design and development of interventions and care models which will achieve high uptake, and therefore, provide a population-level benefit. Consumer perspectives for socially disadvantaged or indigenous groups such as Aboriginal Australians are yet to be explored. Health behaviours among such groups reflect differences in broad influences such as the social environment [26]. For example, smoking is a largely shared and normalised behaviour for many disadvantaged and indigenous communities [27, 28]. Similarly the need for physical activity to be communal or family-oriented, rather than done for the individual alone, has been reported for indigenous populations from Australia [29] and the US [30]. Therefore, tailored approaches to addressing risk behaviours may be needed. Health promotion and prevention strategies aimed at the general population may be less effective in high-risk communities such as Indigenous communities [31], for reasons including the appropriateness of services and support offered [28], use of inappropriate language and messages [31], and limited access to care [32]. A recent review found that culturally enhanced interventions produced better health outcomes than non-enhanced interventions or usual care, for conditions such as diabetes [33]. Given the different social and cultural influences on health risk behaviours [34], generally poorer access to care and lower levels of health literacy [26] of Indigenous and other socially disadvantaged groups compared to less disadvantaged groups, it is critical to gain an understanding of how behavior change might best be supported in these communities.

### Need to assess preferences and priorities for health behaviour change

Behavioural medicine literature suggests that people are more likely to achieve behaviour change when they actively participate in the choice of change to be made [35, 36]. Although individual priorities for change may not reflect the risk posed by the behaviour, they reflect perceptions of likely success, confidence, what might be least difficult to change, and readiness to change [36–38]. Stage of change models provide one way to assess an individual’s readiness to change [39]. Although the evidence is not overwhelming [40, 41], interventions matched to stage of change have shown promise for improving behaviours [37, 39]. Intention to change is generally agreed to be among the most proximal influences over behaviour [42], and a lack of intention generally leads to lack of behaviour [43]. While intention does not guarantee action, stages of change are nonetheless important in predicting the likelihood of subsequent changes in behaviour [44]. Of particular relevance to multiple heath behaviour change approaches is evidence suggesting success with changing one behaviour enhances motivation or readiness to change additional behaviours [13].

The development of culturally targeted health interventions addressing some of the major risk factors for socially disadvantaged groups such as Aboriginal Australians would therefore benefit from a better understanding of priorities for behaviour change, readiness to change, and preferences for types of support. The aims of this study were to explore, among participants attending a primary health care service targeting an Aboriginal Community:

- Readiness to change at risk health behaviours including overweight, smoking, risky alcohol intake, drug use, physical inactivity, poor diet, and depression;
- The acceptability of addressing multiple risk factors independently, sequentially or simultaneously;
- The types of support services which would be used to help participants to change risky behaviours, and whether services should be offered to individuals alone, or to individuals as well as their support person or wider support network; and
- Any significant socio-demographic predictors of stage of change and acceptability of making multiple health behaviour changes.

## Methods

### Setting and participants

An anonymous, cross-sectional health risk survey was administered on a touch screen laptop to people attending a large Aboriginal Community Controlled Health Service (ACCHS) in regional New South Wales (NSW) [45]. ACCHSs provide the majority of comprehensive primary health care to Australian Aboriginal communities [46], with approximately 75–85 % of people attending these services being of Aboriginal or Torres Strait Islander origin [47, 48]. ACCHSs also play a role in community support, special needs programs and advocacy [49]. Informed consent was sought from all participants in the study. Ethics approval for the research was obtained from the University of Newcastle (reference: H-2011-0153) and the Aboriginal Health and Medical Research Council of NSW (reference: 806/11). The study adhered to STROBE guidelines [50].

### Participants and procedure

General details of the study procedure have been reported elsewhere [51]. Briefly, Aboriginal^1^ and non-Aboriginal adults (≥18 years) attending the ACCHS for a general practitioner (GP) appointment were invited to complete a questionnaire in the waiting room while waiting for their appointment. An Aboriginal Research Assistant (RA) undertook patient recruitment for half of the recruitment period of 2 months in 2013 (with a non-Aboriginal RA undertaking the remaining recruitment). Participants were able to exit the survey if called in for their appointment.

### Measures

The touch screen questionnaire included demographic questions and standardised or validated items assessing health risk status including: body mass index (BMI), smoking, alcohol consumption, level of physical activity, consumption of fruit and vegetables, alcohol intake, drug use, depression and adherence with screening guidelines. Participant weight and height were measured. Current national guidelines or established cut-off scores were used to classify participants as at risk. Details of the measures and cut-offs used to assess risk are included in Additional file 1: Table S1.

A series of questions (presented in Table 1) assessed preferences and priorities for health behaviour change, including stage of change or willingness to change each of the health risks identified above. Participants were asked about any health changes they wanted to make, regardless of their individual risk status. Given the focus on assessing perceptions of multiple health behaviour change, participants were asked specifically about whether they would try to make several health changes at once or one at a time, the support services they would use for making these types of changes, and whether services should be offered to individuals or to individuals as well as members of their wider support network including family or friends. To examine whether particular subgroups might be more or less open to multiple health behaviour change approaches, sociodemographic predictors of willingness to change and preferences for making single or multiple health behaviour changes were also explored.

**Table 1:** Survey items assessing participant priorities for change and preferences for types of support

Item	Response options
1. If you could get help, are there any of these changes you would like to make?	Lose weight Stop or cut down smoking Drink less alcohol Get more exercise Eat more fruit and veg Stop or cut down on drug use Find ways to feel less sad or depressed None of these < *skip to end*>
2. When do you think you will try to < *INSERT response/s from Q1* > ?	I’m already trying to < *INSERT response/s from Q1* > ^a^ In the next month In the next 2–6 months Sometime, but not in the next 6 months
3. If you could get help (e.g. from your doctor or a health worker), what would be the best way for you to make these changes? <*For those selecting two or more changes in Q1*>	I would finish making one change before I started on the next one Once I started to get somewhere with one change, I would start on the next one I would try to make some or all of these changes at the same time
4. Would you use any of these services to help you make this health change (or changes)? <*For those selecting at least one change in Q1*>	Advice and help from my doctor or Health Worker, who checks how I’m going My doctor or Health Worker arranging for me to see a specialist (like a dietician, exercise coach, counsellor) I arrange to see a specialist myself (like a dietician, exercise coach, counsellor) None of these
5. Would you want this help to be: <*For those selecting at least one service in Q4*>	Just for me For me and one support person (like my partner, a parent, sibling or friend) For me and other members of my family or my friends Not sure
6. Would you use any of these services to help you make this health change (or changes)? <*For those selecting at least one change in Q1*>	Go to face to face support group meetings with others also trying to change Use a computer to get emails or on-line advice and support Call a telephone support service for advice and support Take home books or DVDs with information and advice Use a phone app and text messages for advice and support None of these
7. Would you want this help to be: <*For those selecting at least one service in Q4 or 6*>	Just for me For me and one support person (like my partner, a parent, sibling or friend) For me and other members of my family or my friends Not sure

^a^For depression, this response option was “I’m already getting help”

Stages of change were defined following Prochaska [53] and de Vries [13]. ‘Precontemplation’ (unwilling to change) was defined as either not intending to change the behaviour (Q1: “none of these”) or not within the next 6 months (Q2: “Sometime, but not in the next 6 months”). ‘Contemplation/preparation’ (thinking about changing) included those intending to change the behaviour in the next month or next 2 to 6 months (Q2: “In the next month/ In the next 2–6 months”), and ‘action’ (attempting to change) as those currently changing their behaviour (Q2: “I’m already trying to change…”). Maintenance of behaviour change was not assessed.

### Analysis

Any data considered likely to be incorrect for weight (<35 kg or >200 kg) or height (<145 cm or >200 cm) were replaced with missing values. The number of multiple risk factors was calculated for each participant by adding their number of single risks. Chi-square analysis was used to compare the characteristics of consenting and non-consenting participants, and simple proportions used to describe readiness to change, preferences for types of support and other study variables. Any significant predictors of stage of change, acceptability of making multiple health behaviour changes, and preferences for support offered to individuals or wider networks were explored using logistic regression. Predictor variables were gender, age, highest level of education and number of multiple risk factors. A sample size of 200 participants enabled the prevalence of most outcomes to be calculated at the 95 % confidence level with a confidence interval of +/−7 %.

## Results

### Participants and consent rate

Of 367 participants approached, 245 (67 %) consented to participate. There were no significant differences in the age, gender or Indigenous status of study consenters and non-consenters (all *p*’s > .05; data not shown). A total of 211 participants completed at least one of the health risk preference questions and were included in analysis. Other participants were called in for their appointment before completing the survey. The demographics of the sample are shown in Table 2. The main source of income for 66 % of the sample was Centrelink (government welfare). Educational levels were also low compared to the general population [54, 55], indicating the majority of the sample experienced relative socioeconomic disadvantage.

**Table 2:** Demographic characteristics of the study sample (*n* = 211)

Demographics	n (%)
Sex	
Male	83 (39 %)
Female	128 (61 %)
Age	
18–24 years	22 (10 %)
25–34 years	44 (21 %)
35–44 years	39 (19 %)
45–54 years	45 (21 %)
55–64 years	46 (22 %)
65 years+	15 (7 %)
Aboriginal status (*n* = 210)	
Aboriginal	182 (87 %)
non-Aboriginal	28 (13 %)
Highest education level	
Primary school	4 (2 %)
Year 10 or below	118 (56 %)
Year 12/TAFE	53 (25 %)
University/ other tertiary	34 (16 %)
Other	2 (1 %)
Income source	
Centrelink	139 (66 %)
Part time/casual employment	20 (10 %)
Full time employment/ self employed	46 (22 %)
Other	6 (3 %)

### Prevalence of risk factors

**Fig. 1:**
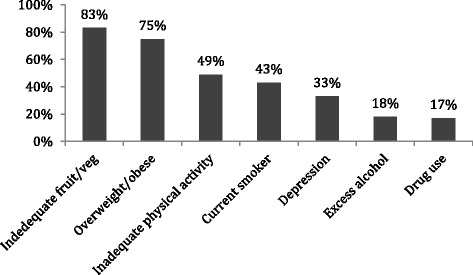
Proportion of sample classified as at risk for each of the health risk factors assessed in the survey

The prevalence of each risk factor for those with complete data is shown in Fig. 1.

### Readiness to change health behaviours

Table 3 shows the number of participants who indicated that they wanted to make at least one of the health changes in the survey, and their stage of change, as a proportion of the total number of participants classified as at risk for each factor.

**Table 3:** Number (percentage) of at risk participants wanting to make one of the survey health changes and their stage of change

Risk factor (n at risk)	n (%)^a^ wanting to change [95 % CI]^a^	Stage of change n (%)^a^ [95 % CI]^a^		
		“Already trying”	In next 1–6 months	“Sometime in the future”
Low fruit/veg intake (*n* = 170)	39 (23 %) [17, 29 %]	32 (18 %) [13, 25 %]	6 (4 %) [1, 7 %]	1 (0.5 %) -
Overweight (*n* = 150)	76 (51 %) [43, 59 %]	56 (37 %) [30, 45 %]	15 (10 %) [5, 15 %]	5 (3 %) [4, 6 %]
Inadequate exercise (*n* = 100)	38 (38 %) [28, 48 %]	19 (19 %) [11, 27 %]	15 (15 %) [8, 22 %]	4 (4 %) [0, 8 %]
Smoking (*n* = 89)	62 (69 %) [60, 79 %]	37 (42 %) [31, 52 %]	14 (16 %) [8, 23 %]	11 (12 %) [5, 19 %]
Depression (*n* = 69)	27 (39 %) [27, 51 %]	14 (20 %) [10, 30 %]	9 (13 %) [6, 27 %]	4 (6 %) [1, 11 %]
Excess alcohol (*n* = 37)	13 (35 %) [19, 51 %]	9 (24 %) [10, 39 %]	1 (3 %) [−3, 8 %]	3 (8 %) [−1, 17 %]
Drug use (*n* = 32)	14 (44 %) [26, 62 %]	10 (31 %) [14, 48 %]	1 (3 %) [−3, 9 %]	3 (9 %) [−1, 20 %]

^a^As a proportion of the total number of participants who were classified as at risk for each factor

As shown, smoking had the highest proportion of those at risk wanting to change, with 69 % of current smokers wanting to stop or cut down, and 52 % already trying; followed by weight, with 51 % of those who were overweight or obese wanting to lose weight, and 37 % already trying to do so. The lowest proportion was for those classified as at risk with poor diet, where 23 % indicated wanting to eat more fruit and vegetables and 18 % reported already trying to do this.

For at risk participants across all behaviours, the only significant predictor of readiness to change was the number of multiple risk factors. Those with a greater number of risk factors were more likely to be in the contemplation (already trying to change or wanting to change in the next 1–6 months) than the precontemplation stage (OR = 1.71, SE = 0.30, *p* = 0.002). Age, gender and level of education were not related to readiness to change.

### Acceptability of multiple behaviour change interventions

All survey participants (*n* = 207) had at least one risk factor, with 94 % having two or more and 67 % three or more multiple risk factors. Approximately half of participants (51 %) reported that there was a single health change that they wanted to make, while 34 % reported wanting to make two or more health changes, and 15 % no health changes.

Of those participants who were contemplating more than one health change (*n* = 68; data missing for *n* = 2), 44 % indicated that they would make one change at a time, 32 % chose overlapping changes (‘once I started to get somewhere with one change I would start on the next’), and 24 % indicated they would try to make some or all of the selected health changes at once. None of the selected variables (gender, age, level of education or number of multiple risk factors) were significant predictors of wanting to make changes at once versus one at a time.

### Types of support wanted

Any participant who indicated there was at least one health change they wanted to make (*n* = 176) was asked to select the types of support or help they would use to help them make these changes. The types of support chosen, as a proportion of those selecting at least one health change, are shown in Table 4.

**Table 4:** Number (percentage) of participants selecting each type of support for participants wanting to make at least one health change

Type of support	n (% [95 % CI])^a,b^
Advice and help from doctor/Health Worker	96 (55 % [47, 62])
My doctor/Health Worker arranges for me to see a specialist	65 (37 % [30, 44])
I arrange to see a specialist myself	19 (11 % [6, 15])
Face to face support group	86 (49 % [42, 57])
Computer emails and online support	22 (13 % [7, 17])
Telephone support service	26 (15 % [10, 20])
Books or DVDs	30 (17 % [12, 23])
Phone ‘app’	12 (7 % [3,11])
None of these	21 (12 % [7,18])

^a^As a percentage of the number of participants who wanted to make at least one health change

^b^Percentages do not add to 100 % as participants could choose more than one type of support

The most common health changes that participants would use advice and support from their GP for included losing weight, getting more exercise, and improving diet. Similarly, face to face support groups would most commonly be used for losing weight, getting more exercise, improving diet and smoking cessation (data not shown).

### Support for individuals only or including family and friends

For participants who chose one of the first three types of support (those related to GP or specialist care; *n* = 146), the majority indicated that they would like this help to be just for themselves (62 %) and about one-third (35 %) for themselves plus a support person, or other members of their family or friends; with 3 % not sure. For participants choosing one of the latter non-clinical types of support (such as support groups or DVDs; *n* = 128), about half wanted this support to be for themselves only (52 %), or for themselves as well as a support person or other members of their family or friends (45 %), with 3 % were not sure.

## Discussion

There was a high prevalence of risk behaviours among our sample, including two-thirds of participants being overweight or having a poor diet, and almost half being physically inactive or current smokers. This is in line with national data for Indigenous Australians [16], and other indigenous and disadvantaged populations internationally [15, 56]. There was a high degree of readiness to change some behaviours, in particular smoking and being overweight. In contrast, few at risk participants were contemplating reducing their alcohol consumption, or increasing their physical activity or fruit and vegetable intake. Of note, the latter two behaviours were among the most highly prevalent, but were associated with the least willingness to change. The vast majority (94 %) of the sample had multiple health risks, but under half reported wanted to change more than one risk factor. Of these, more people preferred the idea of addressing one risk at a time than making more than one change at once. Face to face support services were the most likely to be used, while electronic approaches (such as smart phone apps or websites) were the least popular for making these health changes. Clinical based services (such as GP, specialist) were generally seen as appropriate for individuals, while services such as support groups and educational materials were often preferred to be available to individuals and their wider support network, including a support person or family members and friends.

The majority of smokers in our sample were contemplating or actively trying to quit or cut down (69 %), which is broadly similar to findings for Indigenous Australian smokers from remote Northern Territory communities (58 % contemplating and 17 % attempting to quit) [57] and for women attending an Aboriginal Health Service for ante- or post-natal care (55 % contemplating and 13 % attempting to quit) [58]. Similar rates of cutting down or attempting to quit have been reported for First Nations communities in Canada (46 %; [59]). In contrast to our results, higher proportions of a sample of at risk urban Australian Aboriginal adults were contemplating increased fruit intake (76 %) and physical activity (80 %), compared to increasing vegetable intake (46 %) or smoking cessation (23 %) [7], for reasons which are not clear.

Our sample showed a general lack of readiness to change diet, increase physical activity and reduce alcohol consumption. Less than a quarter of those with a poor diet reported wanting to eat more fruit and vegetables, which has implications for the 51 % of overweight participants reporting wanting to lose weight (although other dietary changes, such as reducing fat intake, were not assessed). Low motivation to change may be due to a lack of awareness of the risks associated with poor diet, physical inactivity or excess alcohol. Over half of the participants from a survey of Aboriginal organisation employees self-reported that they had a ‘healthy diet’, despite half not eating vegetables, and 66 % not eating fruit, on a daily basis [60]. The majority of respondents (72 %) believed that they “…already eat enough [fruit and vegetables]”. Similarly for alcohol, participants in an urban Aboriginal community were not aware of current or previous drinking guidelines and expressed surprise at the low recommended limits [61].

Our sample also showed a general reluctance to address multiple risk factors despite almost all participants having multiple risks. For the 34 % of this sample who indicated that there were multiple health changes they wanted to make, just over half indicated that they would be willing to make some or all of these changes at the same time, or to start on one change and move on to another once they started to get somewhere with the first. No other work exploring preferences for making health changes sequentially or simultaneously for Aboriginal Australians or other socially disadvantaged groups was identified. The most acceptable approach to multiple health behaviour change for this population is likely to be a sequential one in which individuals are able to choose an initial target behaviour. Such an approach may also be feasible for those people reporting only wanting to make one change. For example, success in changing one gateway behaviour may provide increased motivation and self-confidence to attempt more difficult changes [62]. Although individuals may not choose to start with the highest priority behaviour in terms of health benefit, tailoring interventions to individual priorities or readiness may result in greater success in achieving at least one behaviour change, which may in turn increase motivation and confidence to address additional behaviours [37].

The most popular types of support for those contemplating single or multiple health changes included advice and support from a GP or Health Worker, face to face support groups, and seeing a specialist. Support that would be least likely utilised included smart phone apps, web based approaches and telephone support. These preferences also reflect those reported for other Aboriginal communities and socially disadvantaged groups. For example, face to face counselling or group support was preferred for physical activity and smoking among an urban Australian Aboriginal community [7], and few Aboriginal respondents indicated that they would use written materials or telephone support for addressing alcohol problems [61]. Disadvantaged inner city mothers in the UK preferred home visits to telephone for postnatal support [63]. Despite this, there is some evidence that telephone or electronic based approaches can be effective for lower income or indigenous groups [64–66]. These findings have major implications for primary care services, as those preferred types of support are the most costly and time intensive options to deliver. The electronic delivery of support (e.g. via internet or smart phone) would require additional efforts to ensure uptake or access. If not equally accessed or adopted, electronic support initiatives could potentially exacerbate existing health inequalities for already disadvantaged groups.

To our knowledge, no previous studies have explored Aboriginal community preferences for including support persons, family or friends in support strategies for behaviour change. Our results suggest that clinically delivered support is acceptable on an individual level, while community oriented services such as support groups and educational materials would benefit from including a wider network of close family and/or friends. Lack of family support and sense of social isolation have been reported as significant barriers to dietary change for Aboriginal Australians [67]; while some types of physical activity, such as solitary exercise, or done for the benefit of the individual, were associated with feelings of shame or disconnection from others [29]. These principles are likely to apply to other disadvantaged and indigenous groups where, for example, the social environment has also been recognised as a key influence on behaviours [28, 30]. It is therefore likely to be important to offer individuals the choice of having other support persons, including family or close friends, participate in health behaviour change support services or interventions, particularly for those services offered in addition to clinical interactions.

### Limitations

Several study limitations should be noted. Firstly our sample was small and drawn from one ACCHS in an inner regional location, which may limit the generalisability of our results, such as to Aboriginal Australians living in urban or remote areas. However, a substantial proportion (43 %) of Aboriginal Australians live in inner or outer regional areas [68]. Secondly, self-report data was used to assess risk status, health priorities and support preferences. Although validated measures of risk assessment were used where possible, many show only moderate sensitivity and specificity, and most have not been specifically validated for use with Indigenous Australians. In addition, the cut-offs used for risk status, although based on national guidelines, set a low threshold for classification as at risk (for example, those consuming less than seven serves of fruit and vegetables per day, those who were overweight as well as obese). Finally, preferences or attitudes do not always predict subsequent behaviour [69]. Therefore, preferences indicated in our survey may not necessarily reflect behaviour, were the preferred types or modes of services to be offered. However, preferences hopefully provide, at a minimum, some indication of the likely acceptability of such services in this setting.

### Implications for service delivery

A large proportion of people attending an ACCHS were willing, if not already trying, to make positive changes for their health. Further support services for smoking cessation and weight loss offered in the ACCHSs setting are needed to capitalise on the existing motivation and efforts of people to quit and to lose weight; while efforts to decrease alcohol consumption, and increase fruit and vegetable intake and physical activity may need to first focus on raising awareness of current recommendations and the risks associated with these behaviours. Large benefits may be gained from addressing diet and physical activity, given that these were highly prevalent. GPs or Health Workers were the most preferred source of advice and support for behaviour change, and thus need not feel reluctant in discussing health risk behaviours with clients. Although health care providers need to be aware of the significant stressors and pressures which can potentially drive unhealthy lifestyle choices for indigenous and other socially disadvantaged groups [70], our results indicate this should not be a reason for health care providers to avoid giving health risk advice. The option for support services to include support persons, family members or friends was chosen by a significant proportion of participants and is likely to improve health outcomes.

Given the large proportion of those attending the ACCHS indicating there was only one health change that they wanted to make, a sequential, choice-based strategy within the context of a multiple behaviour change approach may be appropriate for addressing the high prevalence of multiple risks in this population, and other indigenous and socially disadvantaged groups. Initial success with the first choice of behaviour is likely to increase the motivation and confidence of individuals to tackle additional health changes. A long term or stepped care management approach will be needed to ensure that other, potentially more difficult health changes, are kept on the agenda. Preferences for face to face delivery of support services suggest the need for caution with adoption of electronic approaches to health behaviour change, and underscore the need for adequate resourcing of services such as ACCHSs, which provide preventive care for predominantly disadvantaged communities.

## Conclusion

Approaches addressing multiple health behaviour changes are likely to be acceptable in ACCHSs as well as other socially disadvantaged primary care settings. However, such approaches may need to allow individual flexibility in choosing an initial target behaviour or behaviours, in timing of subsequent changes, and in the types and format of support provided.

## Additional files

Additional file 1:
**Table S1.** Includes details of the measures used to assess risk factors in the health risk survey, as well as the cut-offs used to define risk status. (DOCX 16 kb)

## Abbreviations

ACCHS(s): Aboriginal community controlled health service(s)
BMI: Body mass index
GP(s): General practitioner(s)

## Footnotes

1 The term ‘Aboriginal’ is used to refer to the Aboriginal and Torres Strait Islander participants in this study, following the guidelines of the New South Wales Department of Health, in recognition that Aboriginal people are the original inhabitants of NSW [52].
