# Supplementary material for: Ready, set, go: a cross-sectional survey to understand priorities and preferences for multiple health behaviour change in a highly disadvantaged group
Source: BMC Health Serv Res. 2016 Sep 13;16(1):488. doi: 10.1186/s12913-016-1701-2 (PMC5020458; doi:10.1186/s12913-016-1701-2)
Supplement: Additional file 1: — Table S1. Includes details of the measures used to assess risk factors in the health risk survey, as well as the cut-offs used to define risk status. (DOCX 16 kb) [file 12913_2016_1701_MOESM1_ESM.docx]

Table 1: Details of the measures and cut-offs used to assess risk

| **Risk Factor and measures** | **Cut-off used to classify ‘at-risk’** |
| --- | --- |
| **Body Mass Index (BMI)**  Measured height and weight  **Smoking status**  ‘Which of the following best describes your smoking?’  **Risky alcohol use**  Two items based on third question  (AUDIT-3) of the AUDIT-C [1, 2]:  ‘How often do you have more than 2/4 standard drinks in one day/on one occasion?’  **Physical inactivity**  Single item [3]; ‘Do you usually do at least half an hour of moderate or vigorous exercise on five or more days a week?’  **Fruit and Vegetable Consumption**  ‘How many serves of fruit/vegetables do you usually eat each day?’  **Depression**  Version of the Patient Health Questionnaire (PHQ-9) modified for use with Indigenous Australians [4]  **Illicit drug use**  ‘When did you last use any illicit or illegal drugs?’ | BMI ≥ 25 kg/m^2^ (excluding pregnant women) [5]  Current smokers (daily or occasional smokers)  > 2 stand. drinks daily or almost daily; or  > 4 stand. drinks weekly or more often [6]  < 30 mins of exercise on five or more days per week [7]  < two serves of fruit; and/or  < five serves of vegetables daily [8]  PHQ-9 score ≥ 10 [9]  Any drug use in the last 12 months (excluding those who responded ‘prefer not to answer’) |

**References:**

1. Babor TF, Higgins-Biddle JC, Saunders JB, Monteiro MG: **The Alcohol Use Disorders Identification Test: Guidelines for Use in Primary Care (Second Ed)**. In*.*: World Health Organization; 2001.

2. Saunders J, Aasland O, Babor T, de la Fuente J, Grant M: **Development of the Alcohol Use Disorders Identification Test (AUDIT): WHO Collaborative Project on Early Detection of Persons with Harmful Alcohol Consumption--II.** *Addiction* 1993, **88**(6):791-804.

3. Rose S, Elley C, Lawton B, A. D: **A single question reliably identifies physically inactive women in primary care**. *The New Zealand Medical Journal* 2008, **121**(1268):ISSN 1175 8716.

4. Esler D, Johnston F, Thomas D: **The acceptability of a depression screening tool in an urban, Aboriginal comminuty-controlled health service**. *Aust N Z J Public Health* 2007, **31**(3):259-263.

5. **BMI Classification** [<http://apps.who.int/bmi/index.jsp?introPage=intro_3.html>]

6. National Health and Medical Research Council: **Australian Guidelines to reduce health risks from drinking alcohol**. In*.* Canberra: NHMRC; 2009.

7. Department of Health and Aging: **Physical activity guidelines for adults**. *Canberra: DOHA* 2005.

8. National Health and Medical Research Council, Department of Health and Aging: **Dietary guidelines for Australian adults: A guide to healthy eating**. *Canberra: NHMRC* 2005.

9. Kroenke K, Spitzer RL, Williams JB: **The Phq‐9**. *J Gen Intern Med* 2001, **16**(9):606-613.
